# Secondary anti‐viral prophylaxis in solid organ transplant recipients for the prevention of cytomegalovirus relapse: A systematic review and meta‐analysis

**DOI:** 10.1111/tid.14393

**Published:** 2024-10-13

**Authors:** David Moynan, Eibhlin Higgins, Matteo Passerini, Larry J. Prokop, Mohammad Hassan Murad, Raymund R. Razonable

**Affiliations:** ^1^ Department of Infectious Diseases Mater Misericordiae University Hospital Dublin Ireland; ^2^ Department of Infectious Diseases University Hospital Galway Galway Ireland; ^3^ Department of Pathophysiology and Transplantation University of Milano Milan Italy; ^4^ Department of Infectious Diseases ASST FBF SACCO Fatebenefratelli Milan Italy; ^5^ Mayo Clinic Libraries Mayo Clinic Rochester Minnesota USA; ^6^ Evidence‐Based Practice Center Mayo Clinic Rochester Minnesota USA; ^7^ Department of Medicine Division of Public Health Infectious Diseases and Occupational Medicine and the William J. von Liebig Center for Transplantation and Clinical Regeneration Mayo Clinic Rochester Minnesota USA

## Abstract

**Background:**

Cytomegalovirus (CMV) is a significant cause of morbidity and mortality in solid organ transplant recipients (SOTRs). Secondary prophylaxis (SP) is not routinely recommended by guidelines on the management of CMV in SOTR but may be considered in certain higher‐risk situations.

**Methods:**

A comprehensive search of English language publications up to September 2023 was performed. The primary outcome was CMV relapse, defined as the recurrence of DNAemia or disease. Secondary outcomes included graft loss, mortality, and hematological toxicity. Meta‐analysis used the random‐effects model. The study protocol is registered in PROSPERO (no. CRD42022357028).

**Results:**

Six retrospective comparative studies were included. A total of 520/727 (72%) of SOTR received SP with valganciclovir. The meta‐analysis did not demonstrate a significant difference in CMV relapse (odds ratio [OR] 1.15, 95% confidence interval [CI] 0.79–2.63). Heterogeneity between the studies was low (*I* ^2^ = 0%, *p* = 0.57). SP was significantly associated with a reduction in mortality (OR 0.2, 95% CI 0.07–0.54) but not graft loss (OR 0.67, 0.17–2.63). There was no significant difference in CMV relapse among kidney‐specific SOTR (OR 1.38, 95% CI 0.65–2.96).

**Conclusion:**

Evidence from six nonrandomized studies is limited and cannot support a recommendation for or against routine SP in SOTR treated for CMV infection. Awaiting prospective‐controlled trials, the decision about SP should depend on individualized risk‐profile assessments by experienced clinicians.

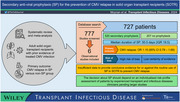

## Graphical abstract

This systematic review and meta‐analysis examining the use of secondary anti‐viral prophylaxis (SP) to prevent cytomegalovirus‐relapse in solid organ transplant recipients does not have sufficient data to provide conclusive evidence for or against its routine use in clinical practice. There is a high degree of bias among the studies included, none of which are randomized controlled trials, and overall contain very low certainty of evidence. We believe this study emphasizes the lack of certainty regarding the efficacy of SP and as such warrants a more robust body of evidence to support its use. Awaiting future larger studies, the decision about SP should depend on an individualized risk‐profile assessment of patients by experienced transplant and infectious diseases clinicians.

**Abbreviations**
CI: confidence interval
CMV: cytomegalovirus
CoE: certainty of evidence
D +: seropositive donor
OR: odds ratio
R –: seronegative recipient
SOTR: solid organ transplant recipient
SP: secondary prophylaxis

## 1 INTRODUCTION

Cytomegalovirus (CMV) is a double‐stranded DNA virus of the herpesvirus family with an estimated seroprevalence of 60%, increasing with age and subject to geographic variability.^1^ Following initial infection, CMV remains dormant within the host and may reactivate during periods of immunosuppression. The reactivation of latent CMV in solid organ transplant recipients (SOTR) is a major cause of morbidity and mortality and as such is a focus of much clinical emphasis.^2^ The risk of CMV reactivation is dictated by the degree of immunosuppression and the serological status of both the transplant recipient and the donor, with a serological mismatch between the donor and recipient conferring the highest risk (CMV seropositive donor and seronegative recipient; CMV D+/R‐).^3^

CMV infection among SOTR manifests in a broad range of clinical presentations ranging from a non‐specific febrile illness to fulminant end‐organ disease including colitis, pneumonitis and hepatitis with the potential for graft rejection and death.^2^ Furthermore, CMV ‘DNAemia’, that is, the detection of DNA in samples of plasma or whole blood, is used as a marker for disease severity and risk of relapse, with a high initial viral load and prolonged DNAemia as risk factors for CMV relapse, occurring in 20–30% of SOTR.^2^ Treatment protocols are established for the antiviral management of CMV disease in SOTR, though strategies for secondary prophylaxis (SP) have not been well defined.^4^, ^5^

SP denotes the use of a prophylactic dose of an antiviral drug after successful treatment of CMV disease in an SOTR with the intention of preventing relapse and maintaining viral suppression.^6^ However, SP is not routinely recommended in international guidance given the lack of solid evidence to support its efficacy, the potential toxicity of antiviral drugs, and the associated cost. As such, while it is a common practice, its safety and usefulness remain largely anecdotal.^4^, ^5^ The practice of implementing SP following the treatment of CMV disease has evolved partly from the use of chronic maintenance therapy in patients with HIV‐associated CMV retinitis and the success of primary prophylaxis in SOTR,^2^, ^7^, ^8^ although there are no randomized controlled trials that demonstrate a benefit in SP in the transplant setting. Given the risk of drug toxicity associated with prophylactic antiviral pharmacotherapy, it is important for clinical evidence to support decision‐making in this regard.

There have only been several single‐center studies that have assessed the outcomes of SP in SOTR with inconsistent outcomes. Here, we performed a systematic review and meta‐analysis to examine the efficacy of SP for the prevention of CMV relapse in SOTR.

## 2 METHODS

### 2.1 Systematic review

#### 2.1.1 Data sources and search strategies

We followed the PRISMA guidelines for systematic review. A comprehensive search of several databases from each database's inception to September 26, 2023, in the English language, was conducted. The databases included Ovid MEDLINE(R) and Epub Ahead of Print, In‐Process & Other Non‐Indexed Citations, and Daily, Ovid EMBASE, Ovid Cochrane Central Register of Controlled Trials, Ovid Cochrane Database of Systematic Reviews, Scopus, and Web of Science. The search strategy was designed and conducted by an experienced librarian with input from the study's principal investigator. Controlled vocabulary supplemented with keywords was used to search for secondary anti‐viral prophylaxis in solid organ transplant patients for prevention of CMV relapse. The strategy listing all search terms used and how they are combined is available in the appendix.

#### 2.1.2 Study selection, data extraction, and quality assessment

Studies eligible for inclusion were required to have a patient population that satisfied the study protocol's inclusion criteria and the domain being studied, that is, a CMV relapse. This was defined as a new CMV infection (classified as DNAemia or disease) in a patient with prior evidence of treated CMV. We included studies encompassing patients (i) aged > or = 18 years, (ii) who received a solid organ transplant, (iii) previously treated for CMV infection, and (iv) with a follow‐up time >6 months after the end of antiviral treatment, acknowledging that 40% will experience relapse within 6 months of stopping antiviral therapy.^9^

Two reviewers (David Moynan and Eibhlin Higgins) screened all titles and abstracts independently using the Covidence platform, a software for managing and streamlining systematic review study analysis.^10^ Studies included at this level by either reviewer were included for full‐text review. Full‐text articles were screened independently by the same reviewers. Disagreements were addressed through discussion and if required, a third reviewer (Matteo Passerini) was consulted with guidance from the principal investigator, as needed. Data were extracted independently by two reviewers including study design, year of study, demographics (gender and age), type of solid organ transplant, CMV serostatus (when documented), type and duration of SP, incidence of CMV relapse, mortality, graft loss and hematological toxicity. Data were inputted into Microsoft Excel v.16.0 for analysis.

The Newcastle‐Ottawa Scale for comparative observational studies was used in the risk of bias assessment.^11^ This examines bias in specific domains. Patient selection, factoring in the representativeness of each cohort and their respective exposure and comparability, was examined in detail. Additionally, outcome measures used, the length of follow, and their adequacy were included in the assessment. Conflicts were resolved through discussion, as above.

#### 2.1.3 Meta‐analysis

Dichotomous data were presented as numbers and percentages. Means and standard deviations, or median and interquartile ranges, were used to describe continuous variables depending on their respective distribution. For the systematic review analysis, the restricted maximum‐likelihood random‐effects model was used owing to the expected heterogeneity of patient characteristics and study setting. Results from the comparative analysis were expressed as an odds ratio (OR) with an associated 95% confidence interval (CI). Meta‐analysis was conducted using Open‐Meta[Analyst].^12^

The certainty of evidence (CoE) was assessed using the GRADE approach. Based on the risk of bias, inconsistency, indirectness, imprecision, or publication bias, the CoE for each study was graded as very low, low, medium, or high.^13^ The protocol of the systematic review is registered in PROSPERO (no. CRD42022357028).

## 3 RESULTS

### 3.1 Systematic review and meta‐analysis

**FIGURE 1:**
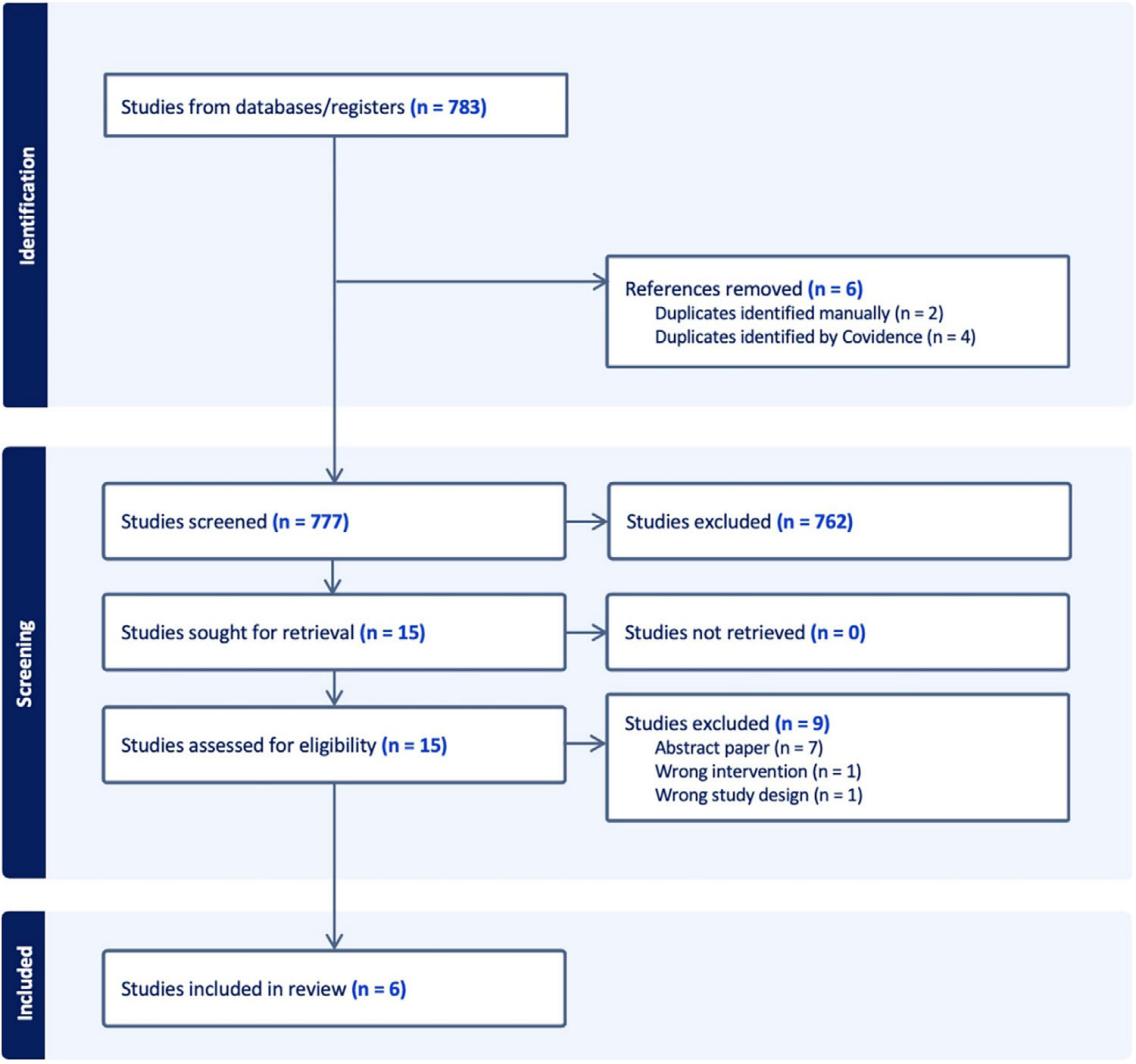
PRISMA flow chart demonstrating the study selection process.

The literature search results are presented in the PRISMA flow diagram in Figure 1. A total of six observational cohort studies were included in the analysis (727 patients; 520 patients receiving SP and 207 not receiving SP).^2^, ^6^, ^14^, ^15^, ^16^, ^17^ The median duration on SP was 50.5 days (interquartile range 19.5 days). All patients received oral valganciclovir at prophylactic doses (or adjusted for renal function) for SP with the exception of the cohort described in Sullivan et al.^14^ who received non‐standardized doses as determined by each treating physician. Successful treatment of prior CMV disease was noted in each paper as a negative viral load by quantitative DNA analysis, by varying methods. High‐risk serostatus (D+/R‐) was noted in 320/727 (44%) of the patient cohort, but the majority of them [219/320 (68%)] received SP. There was no matching data available to include the serostatus in the meta‐analysis. Among the 727 SOTR in the analysis, 208/727 (28%) were kidney transplant recipients, 120/727 (17%) were lung transplant recipients, 91/727 (13%) were liver transplant recipients, 61/727 (8%) were cardiac transplant recipients, and the remainder comprised of a mixed liver‐kidney transplant or otherwise not specified. Demographics are noted in Table 1.^8^, ^12^, ^19^, ^20^, ^21^, ^22^

**TABLE 1:** Table of patient particulars from studies included in the review.

			Secondary prophylaxis				No secondary prophylaxis					
First author	Year	Design	*N*	Male (%)	Age, years, median	CMV D+/R‐, *N* (%)	N	Male (%)	Age, years, median	CMV D+/R‐, *N* (%)	Odds of relapse (95% CI)	Duration of secondary prophylaxis, days, median
Serrano‐Alonso	2018	Observational cohort	103	74	58	14 (14)	21	67	55	3 (14)	0.69 (0.26–1.78)	49
Sullivan	2015	Observational cohort	18	50	N/A	10 (56)	24	67	N/A	17 (71)	1.43 (0.30–6.70)	50
Gardiner	2017	Observational cohort	120	78	53	64 (53)	50	66	52	27 (54)	0.79 (0.37–1.69)	61
Natori	2017	Observational cohort	226	56	55	94 (42)	56	70	54	25 (45)	1.19 (0.37–3.86)	61
Martin‐Gandul	2014	Observational cohort	19	N/A	N/A	19 (100)	28	N/A	NA	28 (100)	1.58 (0.80–3.12)	16
Helantera	2011	Observational cohort	34	N/A	52	18 (53)	28	N/A	52	1 (4)	2.0 (0.64–6.26)	62

### 3.2 Primary outcome: CMV relapse

**FIGURE 2:**
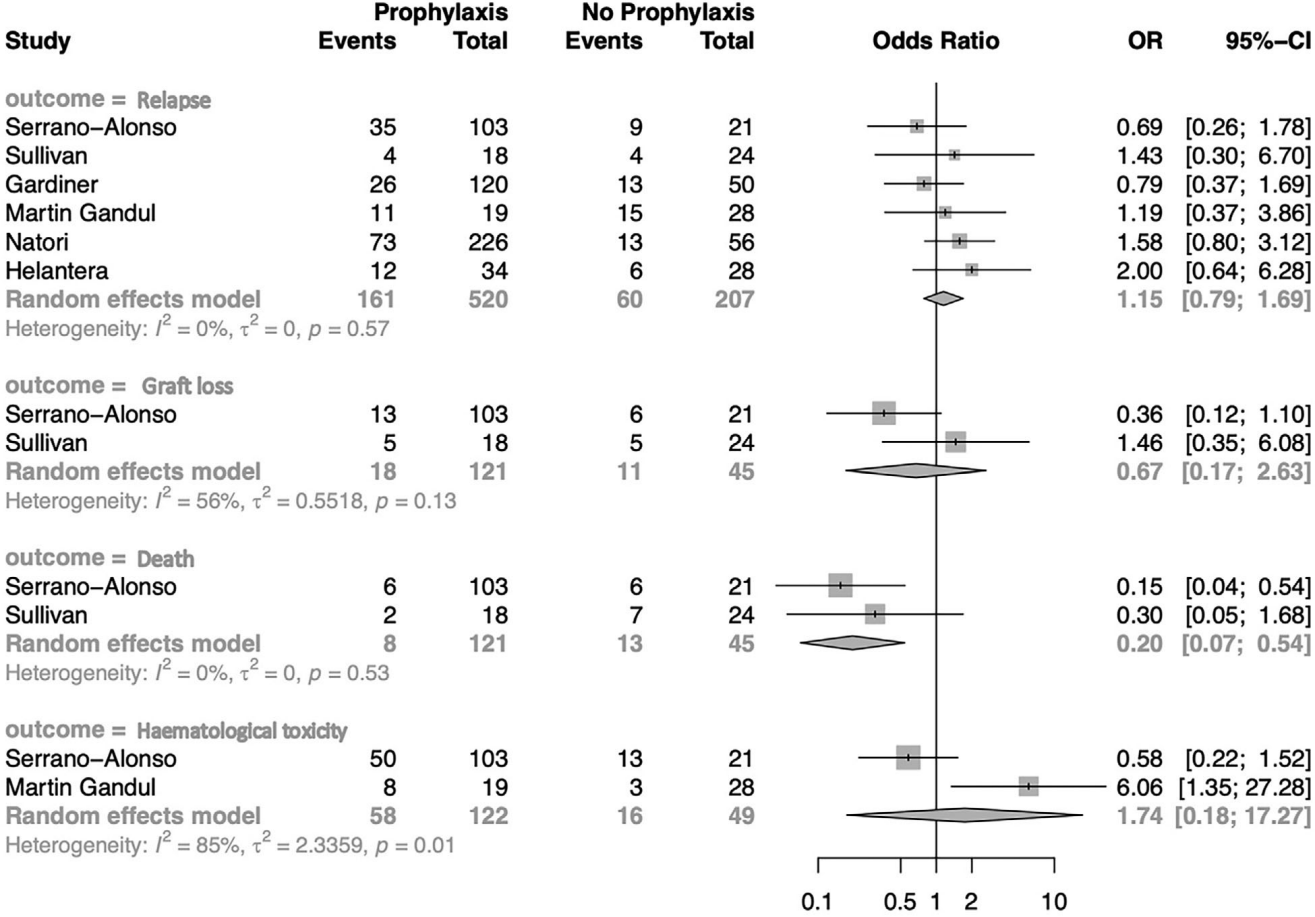
Meta‐analysis of secondary anti‐viral prophylaxis on cytomegalovirus (CMV) relapse, graft loss, death, and hematological toxicity (CI, confidence interval; OR, odds ratio).

**FIGURE 3:**
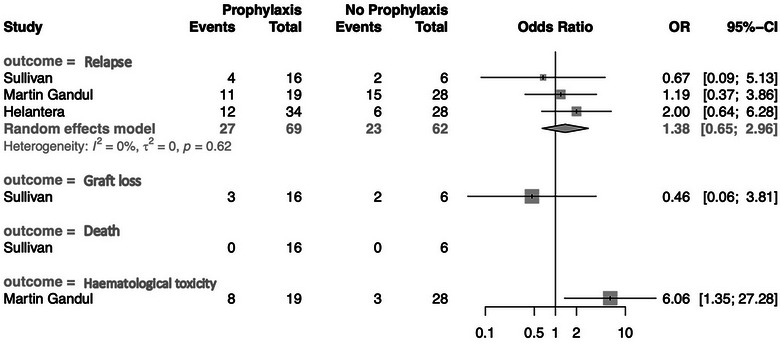
Subgroup analysis examining secondary anti‐viral prophylaxis on cytomegalovirus (CMV) relapse, graft loss, death, and hematological toxicity in kidney‐specific SOTR (CI, confidence interval; OR, odds ratio).

CMV relapse was noted in 161/520 (31%) patients who received SP and 60/207 (29%) patients who did not receive SP, and meta‐analysis did not demonstrate a significant difference between the two groups (OR 1.15; 95% CI 0.79–1.69) (see Figure 2). The heterogeneity between the studies was low (*I* ^2^ = 0%, *p* = 0.57). Outcome data were available in three papers to allow for kidney‐specific metanalyses (*n* = 131). Among the kidney‐specific SOTR, 69/131 (53%) received SP while 62/131 (47%) did not. Analysis restricted to this group failed to demonstrate a significant difference in CMV relapse (OR 1.38; 95% CI 0.65–2.96) (see Figure 3). The estimates of effect were very imprecise, due to the small number of events and warrant very low certainty.

### 3.3 Secondary outcomes

While data were not available in all studies, there was a significant difference in mortality noted, with those receiving SP having a decreased occurrence of death compared to those who did not (OR 0.2; 95% CI 0.07–0.54). Heterogeneity between the studies was low (*I* ^2^ = 0%, *p* = 0.53). There was no difference observed regarding graft loss (0.67; 95% CI 0.17–2.63). Data regarding hematological toxicity were available only in two studies, although among a population of 122/171 who received SP, there was no statistically significant difference in toxicity noted (OR 1.74; 95% CI 0.18–17.27).

### 3.4 Methodological quality of the included studies

All included studies were observational cohorts with no randomized controlled trials. Three of the six studies suffered from a high risk of bias due to the representativeness of the prophylaxis cohort being at the discretion of the prescribing physician and poor comparability among the cohort due to different organ types, genders, ages, and clinical presentation (see appendix 1).^2^, ^15^, ^16^ The remaining three studies were considered a medium risk in their bias with improved comparability in the cohort, however, they again suffered from a high risk of selection bias with the choice of prophylaxis at the physician's discretion.^6^, ^14^, ^17^ The ascertainment of exposure and outcomes was low‐risk overall.

### 3.5 Certainty in the evidence

The CoE for the primary outcome was deemed to be at very low certainty due to the inclusion of only observational cohort studies, of which 3/6 had a high risk of bias and the imprecision of the final result.

## 4 DISCUSSION

This systematic review and meta‐analysis did not demonstrate a significant difference in the rates of CMV relapse among SOTR who received or did not receive SP after treatment of CMV disease. There was a significant reduction in mortality (OR 0.2; 95% CI 0.07–0.54) associated with the use of SP, although the certainty in this estimate was very low, and this reduction in mortality is likely attributed to confounding. Gardiner et al., whose paper demonstrates a mortality benefit following the use of SP, published a supplementary discussion acknowledging the significant confounding factors including level of immunosuppression, illness severity, and age among others.^18^ The current evidence derived from six non‐randomized studies is limited, however, and cannot support a recommendation for or against SP in SOTR.

Our systematic review highlights the limited number of mostly observational data on a common clinical practice of SP in SOTR treated for CMV infection. There are no high‐quality data from RCTs. The VICTOR trial,^19^ a randomized study of oral valganciclovir versus parenteral ganciclovir in the treatment of CMV disease in SOTR, included a maintenance dose of oral valganciclovir in all of its patients following three weeks of standard antiviral treatment. In addition, as noted in the analysis of this study, many of the published studies have small sample sizes and fail to account for significant confounders. Accordingly, our meta‐analysis did not find any significant difference in the rates of CMV relapse between those who did or did not receive SP after treatment of CMV disease.

Regarding toxicity, the meta‐analysis did not find significantly higher odds of hematological toxicity from valganciclovir among the SP cohort, though numbers were low. In order to make a recommendation as to the appropriateness of a pharmacological intervention for patients, safety must be addressed and factored into the decision‐making. In a pharmacokinetic‐pharmacodynamic study including 240 D+/R‐ SOTR receiving oral valganciclovir prophylaxis, the relationship between exposure and myelotoxicity showed a tendency for increased neutropoenia and leukopenia, although admittedly this study did not address concomitant medications which may have potentiated this myelosuppressive effect.^20^ It is possible that the lack of difference in hematological toxicity may be due to the lower dose of valganciclovir for SP compared to treatment doses. Moreover, it may be due to a small number of patients in the analysis; only two of the six studies we analyzed reported on hematological toxicity in their analysis.^6^, ^15^

The heterogeneity in the population included in our study is a significant limiting factor of this review, with a diverse range of serostatus, organ type, and degree of immunosuppression among the patient cohort that cannot be accounted for in the analysis. The risk of acquiring CMV disease in SOTR depends not only on the serostatus of the donor/recipient and the degree of immunosuppression, as noted above, but also on the organ type transplanted. The organ type influences the potency of immunosuppression required, with the highest risk of CMV disease seen in lung and small bowel transplant recipients, known to require a high degree of immunomodulation given the lymphoid tissues transplanted.^21^ While a subgroup analysis of kidney‐specific transplant recipients was performed in an attempt to account for this, serostatus and degree of immunosuppression, including the use of lymphocyte‐depleting agents such as anti‐thymocyte globulin which are associated with an increased risk of CMV disease in SOTR, could not be factored into the analysis. The intensity of pharmacologic immunosuppression also influences the ability of SOTR to reconstitute CMV‐specific cell‐mediated immunity during the treatment of CMV infection.^22^ SOTR with impaired global and CMV‐specific immunity have a higher risk of relapse after treatment of CMV infection.^22^, ^23^

The duration of SP varied significantly between the studies. While there was consistency with regards to the agent of choice for antiviral drug for SP (valganciclovir), the duration varied with Martin‐Gandul et al.^15^ reporting a median of 16 days and Natori et al.^16^ reporting a median duration of 61 days, with a large variation from 5 to 365 days. Furthermore, while the outcome measure chosen in this study was the detection of CMV DNAemia following successful treatment of CMV disease, the clinical manifestations of CMV relapse were broad among the patients included and as such may have influenced the results of mortality on the use of SP in the meta‐analysis. For example, in the study by Gardiner et al.,^2^ there were 39 cases of CMV relapse which varied in presentation, including asymptomatic CMV DNAemia (7/39; 18%), CMV syndrome (17/39; 44%) and end‐organ disease (15/39; 38%). This array of clinical presentations of CMV disease is potentially down to the differing patient characteristics, with some more high risk than others for severe CMV relapse.

In conclusion, given the high degree of bias and the very low CoE in the included studies, there is insufficient data to provide conclusive evidence for or against the routine use of SP in SOTR to prevent CMV relapse after treatment of CMV infection or disease. However, we believe this study emphasizes the lack of certainty regarding the efficacy of SP and as such warrants a more robust body of evidence to support its use, in particular an RCT. The lower odds of mortality among those who received SP are very intriguing and warrant its incorporation as an outcome in future clinical studies. Awaiting future larger studies, the decision about SP should depend on an individualized risk‐profile assessment of patients by experienced transplant and infectious diseases clinicians. Assays that measure CMV‐specific cell‐mediated immunity have emerged to potentially guide the individualized management of SOTR at increased risk of CMV relapse after treatment of CMV disease.

## AUTHOR CONTRIBUTIONS

**David Moynan**: Conceptualization; data curation; investigation; visualization; writing–original draft; and review and editing. **Eibhlin Higgins**: Conceptualization; data curation; investigation; visualization; and writing–review and editing. **Matteo Passerini**: Investigation; validation; and writing–review and editing. **Larry Prokop**: Data curation; methodology; resources; and writing–review and editing. **M. Hassan Murad**: Formal analysis; supervision; validation; and writing–review and editing. **Raymund R. Razonable**: Project administration; supervision; validation; and writing–review and editing.

## CONFLICT OF INTEREST STATEMENT

The authors declare no conflict of interest.

## Supporting information


Supporting Information

## Data Availability

Data are available upon request.
