# Supplementary figures and images for: Secondary anti‐viral prophylaxis in solid organ transplant recipients for the prevention of cytomegalovirus relapse: A systematic review and meta‐analysis
Source: Transpl Infect Dis. 2024 Oct 13;26(6):e14393. doi: 10.1111/tid.14393 (PMC11666880; doi:10.1111/tid.14393)

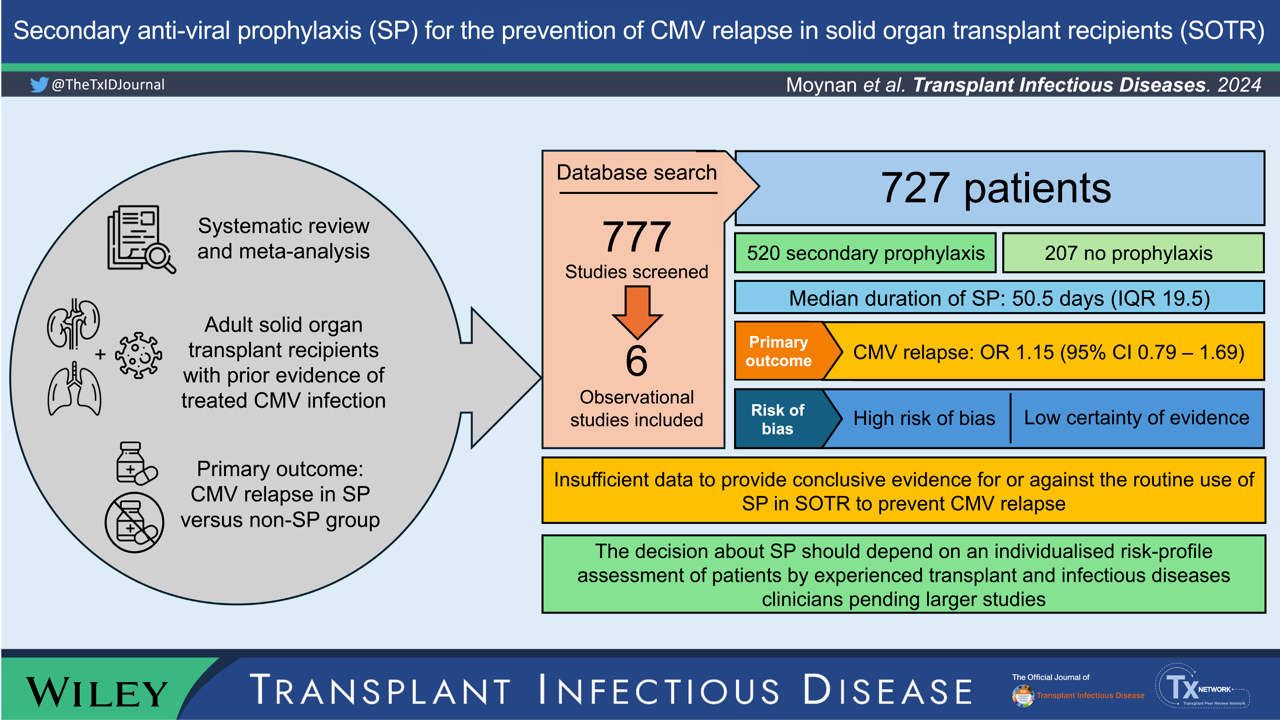

Supplement: Supplementary file 1 — Supporting Information [file TID-26-e14393-s001.tiff]
